# Comparison of Domiciliary and Institutional Delivery-care Practices in Rural Rajasthan, India

**DOI:** 10.3329/jhpn.v27i2.3371

**Published:** 2009-04

**Authors:** Sharad D. Iyengar, Kirti Iyengar, Virendra Suhalka, Kumaril Agarwal

**Affiliations:** Action Research and Training for Health, 772 Fatehpura, Udaipur 313004, Rajasthan, India

**Keywords:** Childbirth, Cross-sectional studies, Emergency care, Fundal pressure, Knowledge, attitudes, and practice, Labour, Obstetric care, Oxytocin, Retrospective studies, Traditional birth attendants, India

## Abstract

A retrospective cross-sectional survey was conducted to assess key practices and costs relating to home- and institutional delivery care in rural Rajasthan, India. One block from each of two sample districts was covered (estimated population–279,132). Field investigators listed women who had delivered in the past three months and contacted them for structured case interview. In total, 1,947 (96%) of 2,031 listed women were successfully interviewed. An average of 2.4 and 1.7 care providers attended each home- and institutional delivery respectively. While 34% of the women delivered in health facilities, modern care providers attended half of all the deliveries. Intramuscular injections, intravenous drips, and abdominal fundal pressure were widely used for hastening delivery in both homes and facilities while post-delivery injections for active management of the third stage were administered to a minority of women in both the venues. Most women were discharged prematurely after institutional delivery, especially by smaller health facilities. The cost of accessing home-delivery care was Rs 379 (US$ 8) while the mean costs in facilities for elective, difficult vaginal deliveries and for caesarean sections were Rs 1,336 (US$ 30), Rs 2,419 (US$ 54), and Rs 11,146 (US$ 248) respectively. Most families took loans at high interest rates to meet these costs. It is concluded that widespread irrational practices by a range of care providers in both homes and facilities can adversely affect women and newborns while inadequate observance of beneficial practices and high costs are likely to reduce the benefits of institutional delivery, especially for the poor. Government health agencies need to strengthen regulation of delivery care and, especially, monitor perinatal outcomes. Family preference for hastening delivery and early discharge also require educational efforts.

## INTRODUCTION

In a rural community of Rajasthan in north India, a qualitative study explored practices by family, community, and care providers during labour and childbirth that might influence health outcomes of newborns (1). A key finding was that multiple persons commonly participated as birth attendants in both home and facility situations. In the home situation, one or two older women, including relatives or traditional birth attendants (TBAs), assumed charge as ‘decision-makers', monitored the entire birthing process, and facilitated delivery. They invited modern care providers, such as nurse-midwives or untrained village practitioners, and modulated their actions and requisitioned help from relatives, neighbours, or the husband for accomplishing ancillary tasks. In primary health facilities, apart from the staff, 1-2 family member(s) or a TBA often accompanied the woman into the labour-room and even helped hasten childbirth by applying abdominal fundal pressure. The study found that, instead of a single birth attendant being in charge of delivery, there often was a team of birth attendants. Whether or not skilled or rational actions were taken at each step during the birthing process depended greatly on the dynamics within the team and on the beliefs and skills of its members. Modern care providers attending delivery in the home conducted frequent vaginal examinations to ascertain progress of labour, applied fundal abdominal pressure, and gave oxytocin injections. Although a doctor or a senior nurse-midwife was in charge in labour-rooms of primary health facilities, the other staff and accompanying relatives or TBAs also carried out pelvic examinations and applied fundal pressure. Local language descriptions of applying fundal pressure (‘to give a hand' or ‘to apply strength') appeared to legitimize its use as a technique to facilitate smooth delivery. To strengthen labour contractions and hasten delivery, modern care providers in home and facility situations administered intramuscular bolus oxytocin (‘heat injections') or (less often) valethamate bromide. Intravenous drips with added oxytocin were popular in health facilities. The family's rationale behind using fundal pressure and heat injections was similar—since women of the younger generation were believed to be weaker and unable to generate the ‘heat' required for forceful labour contractions, delivery had to be facilitated by applying external strength (fundal pressure) and heat (oxytocin) injections.

These findings clearly carried serious implications for maternal and perinatal health in the state. Given the limitations of a qualitative study carried out in two small communities of one district, we decided to validate some key findings by conducting a survey to (a) to assess the frequency of key childbirth practices known to influence maternal and foetal outcomes in rural Rajasthan, (b) compare the roles of care providers attending domiciliary and institutional deliveries, and (c) to estimate care provider-preferences and costs incurred by families seeking maternal and newborn care.

## MATERIALS AND METHODS

We used the Human Development Index of Rajasthan 2001 (2), divided all districts into four quartiles (8 districts each), and purposively selected one district each (Pali and Sawai Madhopur) from the second and third quartiles. We then selected two blocks one from each district with extremes of two criteria, i.e. distance from district headquarters and proportion of population belonging to marginalized groups (scheduled castes and tribes or SC-ST), based on the Census 2001 results. In Pali district, we selected the block that was the closest to the district headquarters (20 km) and had low (25%) SC-ST proportion. In Sawai Madhopur district, we selected a block whose main town was 50 km away from the district headquarters, with higher (38.2%) SC-ST proportion.

We developed a protocol for data collection, drafted, and pre-tested study instruments and then conducted a pilot survey over three days in seven villages of one non-study block of Pali district. A programme for data entry, cleaning, and analysis was developed on the Epi Info software. For the main study, investigators visited each hamlet or cluster of houses in each village (n=221) of the two study blocks, contacted key informants (TBAs, childcare workers, volunteers, and local resident women), and inquired about women who had delivered in the last three months. The list comprised women delivering during June-August 2006 in the first block and during August-October 2006 in the second block. Listing was followed by home-visits to administer structured case interviews to women who recently delivered. A team of eight investigators (4 males and 4 females) were trained over six days, including three days of field training to use these methods, and two supervisors and one research manager supervised them intensively, especially in the first week of field work. The male investigators carried out listing and mapping, and the female investigators conducted case interviews. The field supervisors checked and coded forms in the field and gave daily feedback to the investigators to preserve quality of data. They also observed 15% of interviews by each investigator and separately visited 10% of households to verify selected indicators on the questionnaire from families. Feedback was given to the investigators the same evening.

The investigators took verbal consent for case interviews after providing detailed information on the study objectives and the likely time required. Interviews were conducted in private, unless the respondent was comfortable with the presence of family members. Responses were not shared across families or respondents.

## RESULTS

### Coverage and profile of women

We enumerated 2,031 deliveries over the previous three months in the two blocks (estimated population–279,132). We were unable to interview 84 women (21 families had migrated, 54 houses remained locked even after three visits, and nine women refused consent) and hence, successfully covered 1,947 deliveries (96% of those enumerated) that had resulted in 1,966 births. Assuming similar trends over the entire year, this implied a crude birth rate of 29.1 per 1,000 people which was close to estimates for the state in 2006 (3). Forty-four percent of the women belonged to the scheduled caste and tribal groups. Less than one-fifth of the women were literate, and most had been married off as minors (Table 1). The differences in results for the two blocks were not significant; hence, subsequent analysis based on this criterion has not been presented in this paper.

**Table 1.** Profile of women who recently delivered

Profile of woman	Well- connected block (n=717)	Poorly-connected, interior block (n=1,230)	Total (n=1,947)
Proportion belonging to marginalized scheduled castes and tribes (%)	32.2	50.9	44.0
Female literacy rate (%)	21.5	14.4	17.0
Mean age (years)	25.2	25.4	25.3
Median age at first marriage (years)	16	14	15
Proportion married as minors (aged below 18 years) (%)	71.4	82.7	78.5
Mean age (years) at first cohabitation	17	18	18

### Place of delivery

**Fig. 1.**
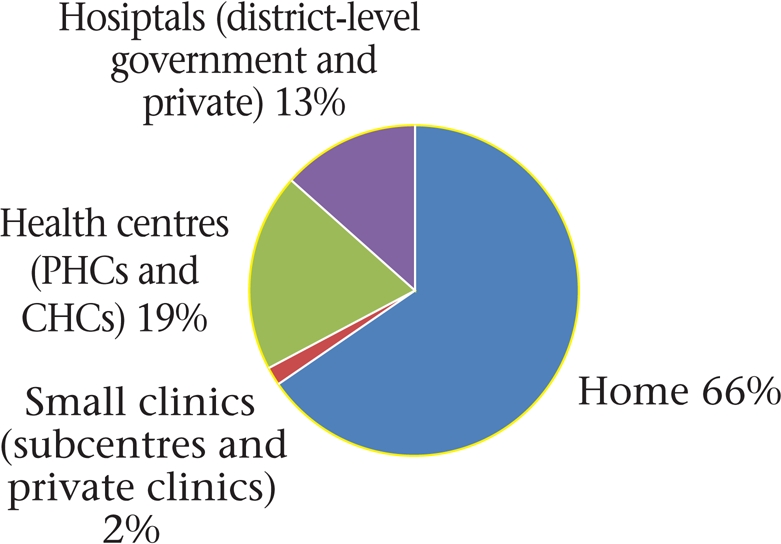
Place of deliveries (n=1,947)

The majority (66%) of the women delivered in the home (Fig. 1). Of those who delivered in an institution, a negligible fraction went to small rural clinics (government subcentres–0.5% and private clinics–1.5%) that lacked proper inpatient facilities. Ten percent went to block-level Community Health Centres (CHCs) that are meant to have multiple doctors, 3-6 nurses, and 30 beds each while 9% went to smaller sector Primary Health Centres (PHCs) that have 1-2 doctor(s), 1-2 nurse(s), and 6 beds each. Another 13% went to hospitals located in district towns, in the government (11%) or private sectors (2%), these being facilities equipped to provide emergency care, including caesarean sections. Most (88%) women had accessed antenatal care at least once, and 88% of them had two or more contacts. While most (98%) women had a vaginal delivery, 39 (2%) underwent caesarean section in the government (n=26) or private (n=13) district-level hospitals. Since only 3.8% of the (1,947) deliveries occurred in private facilities, our results have greater implications for institutional deliveries in the government sector.

CHCs=Community Health Centres; PHCs=Primary Health Centres

**Fig. 2.**
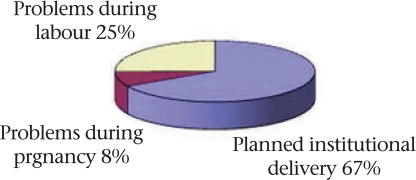
Why did you decide to deliver in an institution? (n=671 woman)

The frequency of institutional delivery declined significantly from 48% for first-order births and 34% for second- and third-order births, to 24% for births of subsequent order (p<0.001). While a smaller proportion of illiterate women and those belonging to the marginalized scheduled castes and tribes opted for institutional delivery, the differences with better-off sections were not significant for vaginal delivery. However, significantly fewer women from scheduled castes and tribes were able to access caesarean section (0.9%) compared to women from other castes (2.8%) (p=0.003). While most women who opted for institutional delivery had made an elective decision well in advance, 33% were either rushed to a hospital because of problems emerging during labour, or because of problems seen earlier during pregnancy (Fig. 2).

### Birth attendants

We defined delivery-care providers as persons who directly participated in examining, monitoring, handling, assisting and/or giving medication to the woman or newborn during labour and till an hour after childbirth. Hence, bystanders were not considered care providers. Modern care providers were those who were trained in western medical tecnniques, or who administered western medicines. They included qualified doctors, female and male nurse-midwives, and unqualified village practitioners and hospical attendants. Half of the deliveries occurred in the presence of a professionally-qualified modern care provider. As expected, most deliveries in the home were managed only by traditional care providers (TBAs and female relatives), and most institutional deliveries were attended solely by modern care providers. Interestingly however, modern care providers had been specifically invited by families to attend a quarter of deliveries in the home while the traditional care providers actively assisted the modern care providers in about one-fifth of institutional deliveries (Table 2). It was also clear that multiple care providers actively assisted in the large majority (74%) of the deliveries, with more being present, on average, during delivery in the home.

**Table 2.** Care providers for delivery (n=1,947 deliveries)

Care provider	Home-delivery (n=1,276)	Institutional delivery (n=671)	Total (n=1,947)
At least one professionally-qualified care provider (doctor/nurse) attended the delivery (%)	24	100	50
Deliveries conducted by single care provider (%)	17	41	26
Mean care providers per delivery	2.4	1.7	2.2
Category of care providers attending (%)			
Only traditional care provider(s)	75.9	0.0	49.6
Traditional and modern care providers	23.7	19.7	22.7
Only modern care providers	0.4	80.2	27.6

### Key delivery practices

We assessed key practices relating to 1,908 vaginal deliveries. Practices relating to 39 caesarean deliveries have been excluded from this analysis. Results on cost of delivery care, however, do include caesarean deliveries.

### Practices to hasten the process of labour

**Fig. 3.**
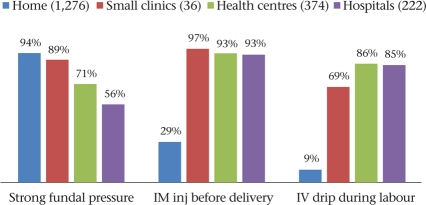
Practices for hastening labour, by venue (1,908 vaginal deliveries)

We assessed three practices that are indicative of efforts to hasten labour—the application of strong abdominal (fundal) pressure, giving of intramuscular injections (generally oxytocin) during labour but before delivery of the baby, and running an intravenous drip during labour. While this retrospective study was unable to pinpoint the nature of injections being given, informal inquiries with healthcare providers and chemist-shops in the area confirmed that oxytocin was the commonest drug being administered both intramuscularly and within drips, with a lesser role for valethamate bromide (epidosin). We found that application of strong fundal pressure was near universal during deliveries in the home and in small clinics (subcentres and private clinics) and was commonly employed in larger health centres and hospitals as well (Fig. 3). Also, most women undergoing institutional delivery received intramuscular injections and intravenous (IV) drips.

IM=Intramuscular; Inj=Injection; IV=Intravenous

During deliveries in the home, strong fundal pressure was applied mainly by TBAs (51%) or female relatives (37%); intramuscular injections were administered mainly by male nurses (15%), Auxiliary Nurse Midwives (ANMs) (6%), or untrained practitioners (2%); and IV drips by male nurses (6%), ANMs (2%), and untrained practitioners (1%). During institutional deliveries, fundal pressure was largely applied by doctors (31%) and/or nurses (31%). Intramuscular injections and IV drips were administered by doctors (48% and 42% respectively) and by nurses (45% and 42% respectively) within health facilities.

**Fig. 4.**
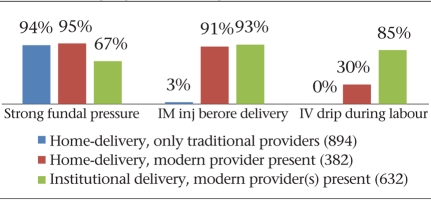
Practices for hastening labour, by provider category (1908 vaginal deliveries)

Fig. 4 shows that the additional presence of modern care providers in home-settings significantly increased the likelihood of intramascular injections and IV drips being used during labour (p<0.001) but made no difference to the use of fundal pressure. Compared to homes, fundal pressure was significantly less likely to be used within health facilities (p<0.001) but was nevertheless used on two-thirds of the women. Even in the presence of a modern care provider, IV drips were used less often during deliveries in the home, probably because setting up an IV line was less feasible in the home situation.

IM=Intramuscular; Inj=Injection; IV=Intravenous

### Episiotomy

Almost a quarter of primigravidae (125 of 513) undergoing vaginal delivery received an episiotomy. The frequency of this practice ranged from 1% in homes, 10% in small clinics, and 32% in health centres, to 72% in hospitals. Thus, episiotomy for primigravidae was far more likely if the woman delivered in a district-level government or private hospital. Among all multigravidae, 7.8% received an episiotomy.

### Practices after delivery

We were able to reliably assess five practices in respect of vaginal deliveries. They included intramuscular injection soon after delivery (which is likely to imply oxytocics to prevent postpartum haemorrhage and is a standard best practice), bathing of the newborn within four hours of delivery, and the giving of prelacteal feeds, both of which are irrational practices (Fig. 6 and 7).

**Fig. 5.**
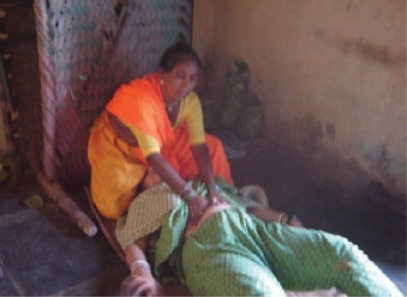
Demonstration of one of the positions of fundal pressure by a traditional birth attendant

**Fig. 6.**
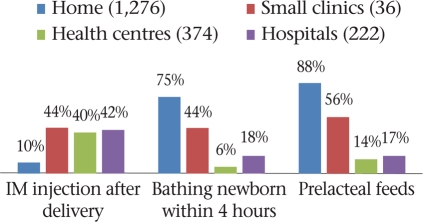
Practices after delivery, by venue (1,908 vaginal deliveries)

IM=Intramuscular

**Fig. 7.**
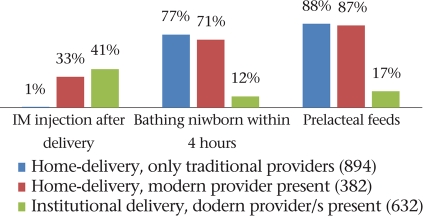
Practices after delivery, by provider (1,908 vaginal deliveries)

IM=Intramuscular

The use of oxytocics after delivery was consistently low across all institutions (41%), even in hospitals (42%). As expected, the presence of a modern care provider during delivery in the home did lead to increased use but only in one-third of the women. These injections were administered mainly by nurses (8%) and village practitioners (2%) in homes and by nurses (23%) or doctors (19%) in institutions.

Early bathing of the newborn was very common in the home-setting (75%), where the additional presence of modern care providers made little difference to curb the practice (71%) (Fig. 6). Early bathing was practised following 12% of institutional deliveries and was surprisingly more common in hospitals than in health centres. While TBAs (39%) and female relatives (35%) bathed the baby in this manner in homes, nurse-midwives (6%) and female relatives (2%) did the same in facilities. The practice of giving prelacteal feeds to the newborn largely occurred in home-settings. It also occurred following 17% of institutional deliveries, implying a lack of attention to initiating breastfeeding, on part of the staff.

A fourth practice that occurred in 31% of deliveries in the home was that of a birth attendant applying the heel of her own foot to the woman's introitus, apparently to restore the tissues to their place and to reduce bleeding. Further, our earlier qualitative study had revealed that, for the first few minutes, domiciliary birth attendants paid attention solely to the placenta and ignored the newborn to the extent that the baby delivered directly onto the floor without someone receiving it. In this study, we found that a mere 11% of newborns in the home were received in the care provider's hands—the rest were allowed to deliver onto the floor.

### Time of discharge after institutional delivery

**Fig. 8.**
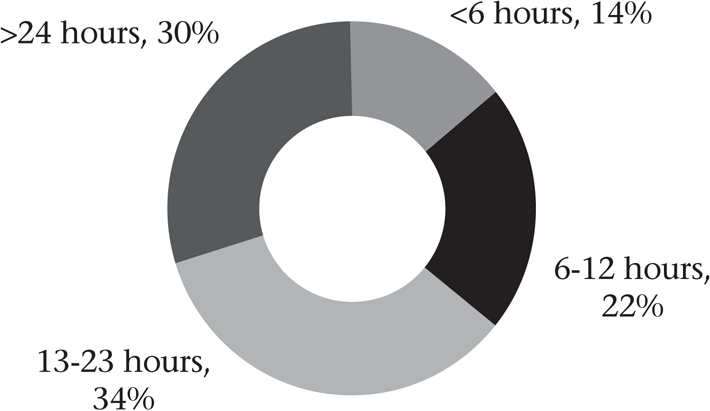
Interval between institutional delivery and discharge (632 vaginal deliveries)

**Fig. 9.**
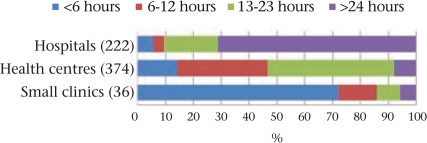
Interval between institutional delivery and discharge, by venue (632 vaginal deliveries)

Sixty-six percent of the women were discharged less than 24 hours after delivery. Since we wished to assess the frequency of premature discharge after routine institutional delivery, we excluded 39 caesarean deliveries from the analysis. Figure 8 shows that 70% of the women undergoing vaginal delivery were discharged prematurely. Results disaggregated by category of facility (Fig. 9) revealed that over 70% of the women delivering in the subcentres and similar small private clinics were sent home within six hours, although the overall numbers were small. The government PHCs and CHCs were able to keep women for longer time but seldom for at least 24 hours. Only in the district-level government and private hospitals did most women stay for at least 24 hours after delivery. Further analysis revealed that rural facilities, such as small clinics and health centres, discharged women earlier than urban facilities (hospitals) (mean 17.9 and 63.2 hours respectively, p<0.01).

### Cost of delivery care

We studied direct medical costs relating to care-seeking (travel, drugs and supplies, laboratory tests, inpatient stay, fees, and tips) and non-medical costs as uncovered during the pilot phase of the study (these related to fulfilling oaths or promises made in anticipation of a good-delivery outcome, rituals including the ‘sun ceremony', and feeding the parturient woman a rich traditional diet with herbal supplements). We further disaggregated the cost of institutional delivery by whether the family had visited the facility electively, or had gone there because of a supervening health problem during pregnancy or labour.

The mean medical cost of deliveries in the home was Rs 379 (US$ 8). Families spent a mean of Rs 1,336 (US$ 30) for elective vaginal delivery in a health facility; this increased to Rs 2,419 (US$ 54) if the woman visited the facility for a problem and had a vaginal delivery. Families reported spending a mean of Rs 11,146 (US$ 248) on caesarean deliveries (Table 3), a large proportion of which was spent on drugs and supplies.

**Table 3.** Mean cost of delivery (Rs) in homes and facilities (1,947 deliveries)

Mean cost	Home-delivery (n=1,276)	Vaginal delivery following elective facility visit (n=429)	Vaginal delivery following facility visit for a problem (n=203)	Caesarean section (n=39)
Medical costs				
Provider fees	261	369	506	2,326
Drugs and supplies	150	325	625	3,690
Tips to staff/providers	108	44	56	190
Travel	8	432	576	881
Room charges	0	16	32	307
Mean medical costs	379	1,336	2,419	11,146
Median medical costs	300	1,030	1,480	10,575
(range)	(0-3,900)	(0-17,800)	(100-21,500)	(0-30,000)
Non-medical costs				
Fulfilment of oaths	278	401	545	253
Ceremonies	528	1,112	703	1,181
Traditional diet with herbal supplements	2,317	2,085	2,172	2,535
Mean non-medical costs	3,112	3,604	3,399	4,167
Median non-medical costs	2,700	2,600	2,650	3,500
(range)	(0-35,000)	(0-51,950)	(0-21,900)	(0-16,900)
Total costs				
Mean total costs	3,491	4,940	5,819	15,313
Median costs	3,050	3,850	4,601	14,800
(range)	(0-35,500)	(100-53,000)	(100-26,000)	(1,100-40,000)

**Fig. 10.**
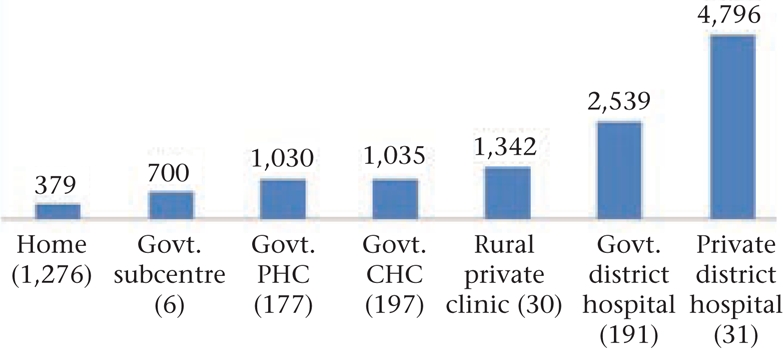
Mean medical cost of vaginal delivery (n=1,908)

Analysis of medical costs by type of institution revealed that, while deliveries in the subcentre cost about twice as much as delivery in the home, rural private clinics staffed by retired paramedics or unqualified practitioners cost even more than PHCs and CHCs (Fig. 10). The cost of vaginal deliveries in government institutions (n=571), on an average, was Rs 1,533 while those in the private facilities (n=61) averaged Rs 3,097. A caesarean delivery in government district hospitals (n=26) cost a mean of Rs 9,075 whereas that in private hospitals (n=13) averaged Rs 15,288. It was clear that, even in government health facilities, fees to care providers and tips to support staff made up a substantial fraction of the overall cost.

CHC=Community Health Centre; Govt.=Government; PHC=Primary Health Centre

In addition to medical costs, families spent a significant amount of money on non-medical expenses. The mean non-medical cost was Rs 3,271, and it did not vary significantly across institution category or type of delivery, although more money was spent following caesarean delivery (Table 3), possibly because most caesarean sections occurred among women belonging to the economically better-off castes.

Over 70% of the families had borrowed money to meet delivery-relating expenses (Table 4). As expected, the mean amount borrowed was greater for institutional vaginal deliveries and was much higher for caesarean sections. While a small proportion of families raising money sold assets, pawned valuables, or borrowed from relatives, the large majority (72%) had to borrow from local money-lenders at mean interest rates of over 2% per month.

**Table 4.** How families raised funds to meet delivery-related expenses (1,947 deliveries)

Indicator	Home-delivery (n=1,276)	Institutional vaginal delivery (n=632)	Caesarean delivery (n=39)
Proportion (%) of families that had to raise funds for delivery	71	76	67
Source of funds (%)			
Sold assets	0.8	0.2	3
Took loan(s)	71	76	64
Mean amounts raised (Rs)	3,076	4,507	14,083
Source of loans (%)			
Relatives	10	8	12
Pawned valuables	2	3	4
Local money-lender	88	89	84
Mean monthly rate of interest	2.1	2.1	2.3

## DISCUSSION

Demographic health surveys across countries have followed a tradition of recording deliveries as having been attended by a single care provider (4-9). Along a hierarchy, the more qualified or professional person is considered to be the birth attendant, in the expectation that s/he would be in charge of the delivery and influence practices. The presence of a skilled attendant during delivery, a key indicator for monitoring achievement of Millenium Development Goal 5 across countries, is meant to imply that the woman and newborn indeed receive skilled care (10). Our study in rural areas of a large north Indian state revealed that more than one person attended the majority of deliveries and that, especially in the home situation, the role of the visiting modern care provider was largely restricted to giving injections or drips during labour. Thus, even in the presence of a professionally-qualified birth attendant, women and newborns were subjected to a range of ‘unskilled' practices in both homes and facilities.

This survey confirmed impressions from the earlier qualitative study that practices to hasten labour are widespread (1). While there are no studies on the effect of fundal pressure during labour, it is expected to increase the possibility of foetal distress, birth trauma, and intracranial haemorrhage (11). Misuse of oxytocin for labour acceleration in home situations has been reported from elsewhere in India (12,13). Intramuscular oxytocin given during labour can result in excessively strong uterine contractions that can trigger foetal distress and birth asphyxia (14). It was evident that, when a modern care provider (nurse-midwife, male paramedic, or unqualified village practitioner) was invited by family members or TBAs to assist during delivery in the home, the likelihood of labour being augmented in a dangerous manner was similar to that occurring in health institutions of the area. In a sense, therefore, families ‘brought home' these features of an institutional delivery by inviting modern care providers.

Following the introduction of a cash benefit scheme—*Janani Suraksha Yojana* (15)—for women delivering in government health facilities, there has been a steep increase in institutional deliveries in several states of India, including Rajasthan (16). Given that commensurate improvements in amenities, staff capacity, and quality assurance within facilities are taking time to introduce, care providers might want to hasten labour so as to ‘free up' labour-tables, maternity beds, and unburden the staff on duty. This has been borne out by our personal communication with doctors and nurse-midwives in three government health centres of the state. On another note, care providers who do not routinely augment labour and follow evidence-based care have reported frequent family pressure on them to ‘do something' to speed up the labour, with some women even having been taken out of the health centre to another care provider who was willing to use injections or drips to hasten labour. The same care providers have also reported that jeep-taxi drivers (who transport women to and from rural health centres for delivery) additionally encourage families to take women back in the same vehicle a few hours after delivery at nominal extra cost (personal communication with nurse-midwives of two ARTH health centres in southern Rajasthan and with senior nurse-midwives of two district hospitals). Hence, it appears that practices to speed up labour might suit the convenience of health staff, families, and transport operators.

Our study also found that actions to prevent postpartum haemorrhage (i.e. active management of the third stage of labour, including injection of an oxytocic) were not up to the standard level—only 43% of women received an injection after delivery. At the time of this survey, a guideline on the use of oral misoprostol for preventing postpartum haemorrhage had been issued (17) but the tablets had not been supplied to facilities. The frequent use of episiotomy for primigravidae delivering in the district-level hospitals suggests a large gap between evidence of effectiveness and practices on the ground in Rajasthan. A Cochrane review has concluded that liberal or routine use of episiotomy had no beneficial effect and was instead associated with increased perineal trauma (18).

Premature discharge after institutional deliveries would not allow the monitoring of the maternal and neonatal condition in the crucial first 24 hours after delivery. In developed countries, the recommended postpartum hospital stay is 48 hours for uncomplicated vaginal delivery (19). Discharge from hospital before 24 hours of delivery has been associated with higher neonatal morbidity and mortality (20-21). State governments in India have recently issued administrative guidelines advising discharge after 24 hours. However, given care provider-preferences and family pressures alluded to above, compliance with the same is likely to be a major challenge.

Given that vaginal delivery and caesarean sections are meant to be provided free to women in government institutions, the reported costs are substantially high and could have played a role in limiting access, especially to caesarean sections for marginalized groups. Although the scheme of cash incentives for institutional deliveries had just started when the study was in progress, families typically had to raise funds for delivery, often by taking loans at high rates of interest. Although traditional postpartum behaviours of women have been documented (22-23), there has been a lack of information on the costs incurred on them. Our observation of significant expenditure on a special diet for the woman, and on rituals and ceremonies can inform communication messages intended to promote the desired postpartum care for mother and newborn.

Every woman has a right to safe delivery, which as a minimum, must include labour monitoring, active management of the third stage, immediate care of the newborn, postpartum monitoring, and prompt action to address complications (24). Our findings suggest that these essential actions do not occur in most home and several facility situations in rural Rajasthan. In addition, the high frequency of irrational acts of commission, such as applying fundal pressure, rampant use of oxytocin during labour, early bathing of the newborn, and early discharge from institutions render deliveries unsafe. Hence, it is crucial that the Government takes serious measures to strengthen its stewardship role by monitoring and regulating delivery-care practices and assesses their likely maternal and perinatal outcomes. Further, it is essential that hidden costs of services at government facilities are minimized if not eliminated so that poor rural families can gain better access. At the same time, campaigns to increase public awareness of these issues might be helpful to apply community pressure to curb these practices. As a demand–side intervention, community-level communication should especially focus on the natural progression of labour and on the need to avoid routinely speeding up the process through the use of manual compression and drugs and to avoid premature discharge.

A limitation of our study was that this was a retrospective survey that relied on women's recall of actions undertaken during labour and delivery, up to three months after delivery. It is possible that recall of some of these actions was not fully accurate. Hence, our findings could be confirmed through surveys with shorter recall periods and observation of practices within facilities and homes. However, given the high levels of non-standard practices reported, it is likely that these practices are commonly undertaken in homes and institutions, by traditional and trained care providers alike.

## ACKNOWLEDGEMENTS

The study was financially supported by the Department for International Development, UK, through the ICDDR,B and Indian Institute of Management, Ahmedbabad. The funders had no involvement in the research, writing, or in the decision to submit the paper for publication.
